# Case report: Fundic gland polyps caused by long-term omeprazole use in a Maltese dog

**DOI:** 10.3389/fvets.2023.1287335

**Published:** 2023-10-23

**Authors:** Haemin Lee, Sanggu Kim, Dohee Lee, Yeon Chae, Taesik Yun, Mhan-Pyo Yang, Byeong-Teck Kang, Soochong Kim, Hakhyun Kim

**Affiliations:** ^1^Laboratory of Veterinary Internal Medicine, College of Veterinary Medicine, Chungbuk National University, Cheongju, Republic of Korea; ^2^Laboratory of Veterinary Pathology and Platelet Signaling, College of Veterinary Medicine, Chungbuk National University, Cheongju, Republic of Korea

**Keywords:** canine, gastrin, gastropathy, proton-pump inhibitor, vomiting

## Abstract

Long-term use of proton-pump inhibitors can induce fundic gland polyps in the human stomach. However, this phenomenon has not been described in the veterinary literature. A 5-year-old intact female Maltese dog was referred to our hospital with chronic intermittent vomiting. The dog had been administered omeprazole (0.7–1.0 mg/kg PO q24 h) for the management of hydrocephalus for over 4 years; the omeprazole dose was increased to 10 mg/kg PO q24 h 8 months prior to presentation at referring hospital. Abdominal ultrasonography revealed marked thickening of the gastric wall with multi-lobulated, thickened folds. Subsequent endoscopy revealed marked polypoid lesions, and histological examination of the biopsy samples was consistent with the fundic gland polyps associated with proton-pump inhibitor use in humans. The lesions resolved after cessation of omeprazole, as assessed by ultrasonography. This report describes a case of fundic gland polyps following the long-term administration of omeprazole in a dog.

## 1. Introduction

Proton-pump inhibitors (PPIs) are commonly prescribed as acid suppressants to treat various diseases in humans and animals. The drugs might also be prescribed for the long-term management of dogs with hydrocephalus (1). Recently, caution regarding the inappropriate or long-term use of PPIs has been raised owing to the potential adverse effects of PPI use without clear indications (2, 3). Most adverse effects of long-term PPI use have been reported in humans, whereas evidence of the effects in dogs is lacking (3).

In humans, histopathological changes in gastric mucosa have been observed following long-term PPI use. These changes are evident on endoscopic examination as fundic gland polyps, hyperplastic polyps, multiple white and flat elevated lesions, cobblestone-like mucosa, or black spots (4). These gastric changes have not been noted in veterinary literature. The use of PPIs with clear indications is underconsideration based on previous studies in veterinary medicine because of the increased risk of adverse effects (5), however, these studies were not focused on long-term PPI use in dogs (6–9). Therefore, the potential adverse effects, including gastric changes with long-term PPI use, are unclear in veterinary medicine, despite the wide use of these drugs. Here, we describe a case of fundic gland polyps that developed after long-term omeprazole administration for the management of hydrocephalus in a dog.

## 2. Case presentation

A 5-year-old intact female Maltese dog weighing 2.5 kg was referred for the investigation of chronic intermittent vomiting for over a year. The dog had been diagnosed with hydrocephalus based on the finding of dilated lateral ventricles on brain magnetic resonance imaging 5 years prior to presentation at our hospital and had been treated with furosemide (1 mg/kg PO q12 h) and omeprazole (0.7–1.0 mg/kg PO q24 h) to reduce the cerebrospinal fluid volume. The administration of omeprazole was continued at the original dose until the dog showed more frequent generalized tonic–clonic seizures 4 years later, when the dose was increased to 10 mg/kg PO q24 h. This dose was administered for 8 months until neurological signs were observed more frequently. At presentation, the frequency of seizures had decreased, but the owner reported that the frequency of vomiting had recently increased.

**Figure 1:**
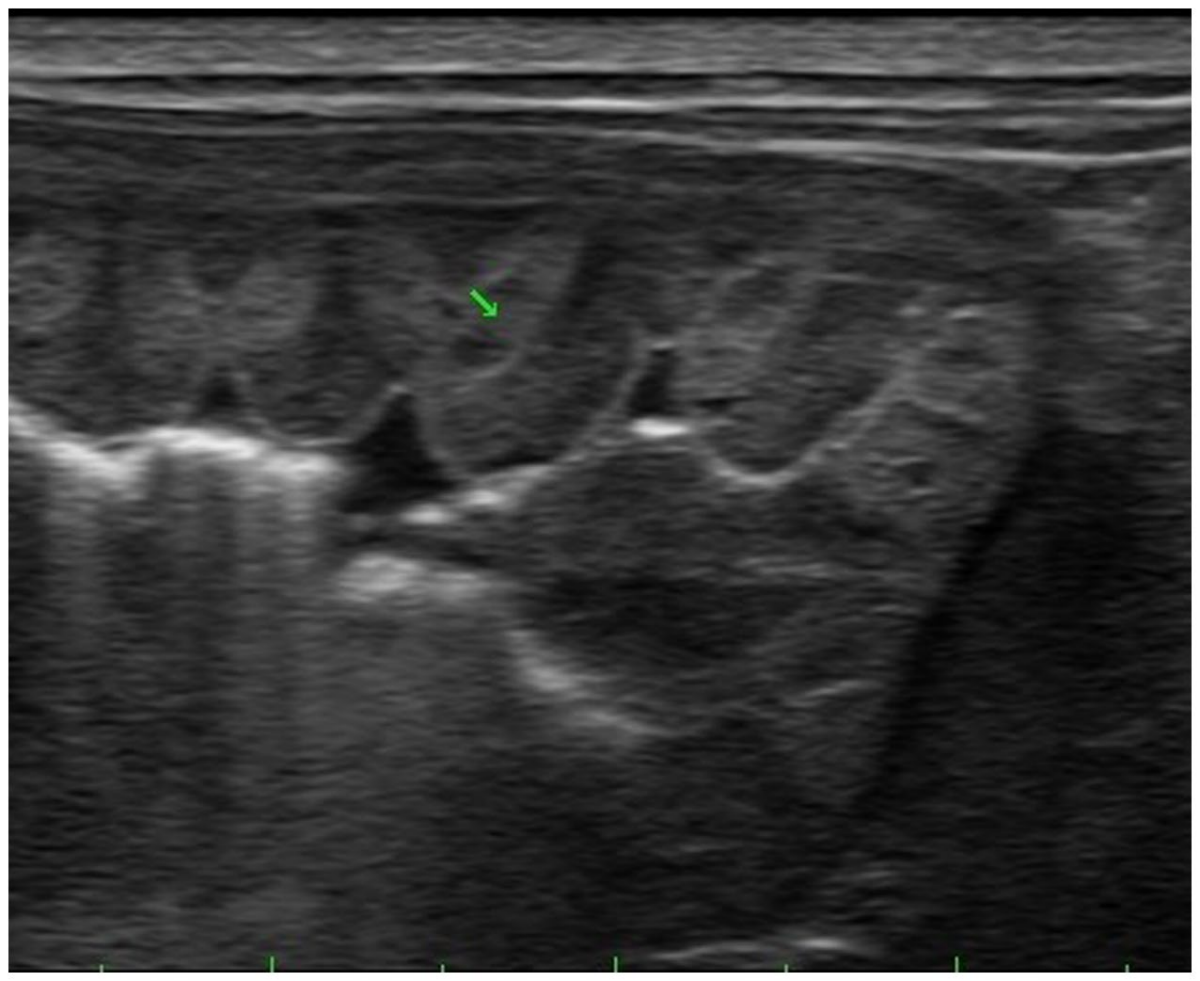
Ultrasonographic view of the stomach, showing mucosal thickening and anechoic cysts in the gastric wall.

On physical examination, the dog was lethargic, had a normal body condition score (3/9), and was tachycardic (> 180 bpm); however, the other vital signs were normal. The oral mucous membrane was pink, but tacky, with a capillary refill time of <2 s. No heart murmurs or adventitious lung sounds were detected during thoracic auscultation. No abdominal pain or abnormalities were detected on palpation, and the results of a brief neurological examination were unremarkable. These findings did not suggest the presence of high intracranial pressure. No abnormalities were observed in the complete blood count or serum electrolyte concentrations. Biochemical analyses revealed high blood urea nitrogen level (33.7 mg/dL; reference interval [RI], 7–28 mg/dL). The survey radiographs were unremarkable. Abdominal ultrasonography revealed marked thickening of the gastric wall, with multi-lobulated, thickened folds, and anechoic cysts. The gastric wall layers were not distinguishable, and the gastric mucosa appeared hyperechoic with no evidence of lymphadenopathy (Figure 1).

**Figure 2:**
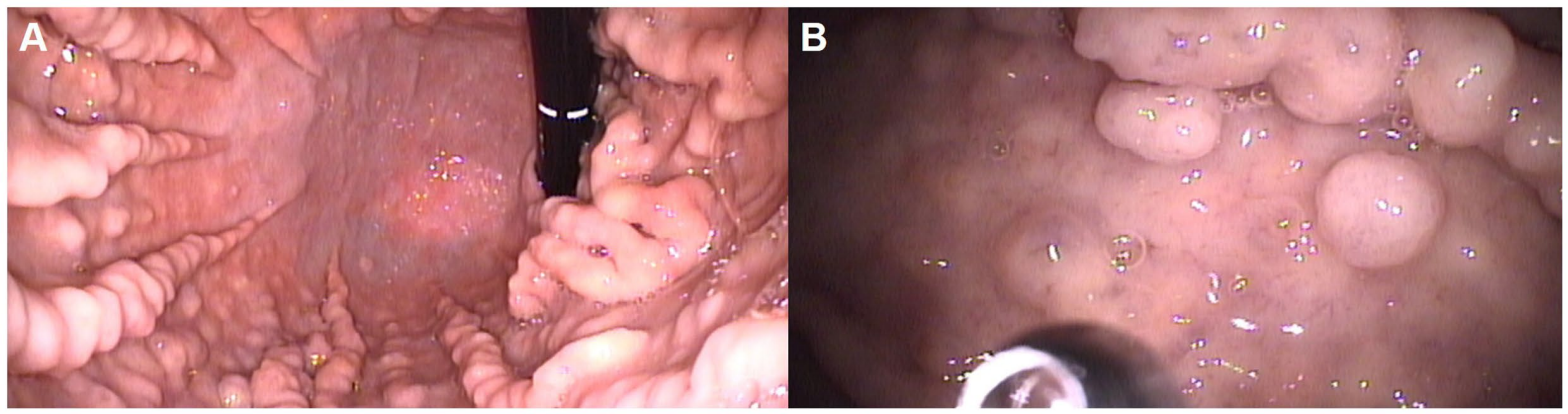
Endoscopic views of the stomach, showing hypertrophied gastric folds **(A)** and a polypoid lesion **(B)** affecting the gastric body.

**Figure 3:**
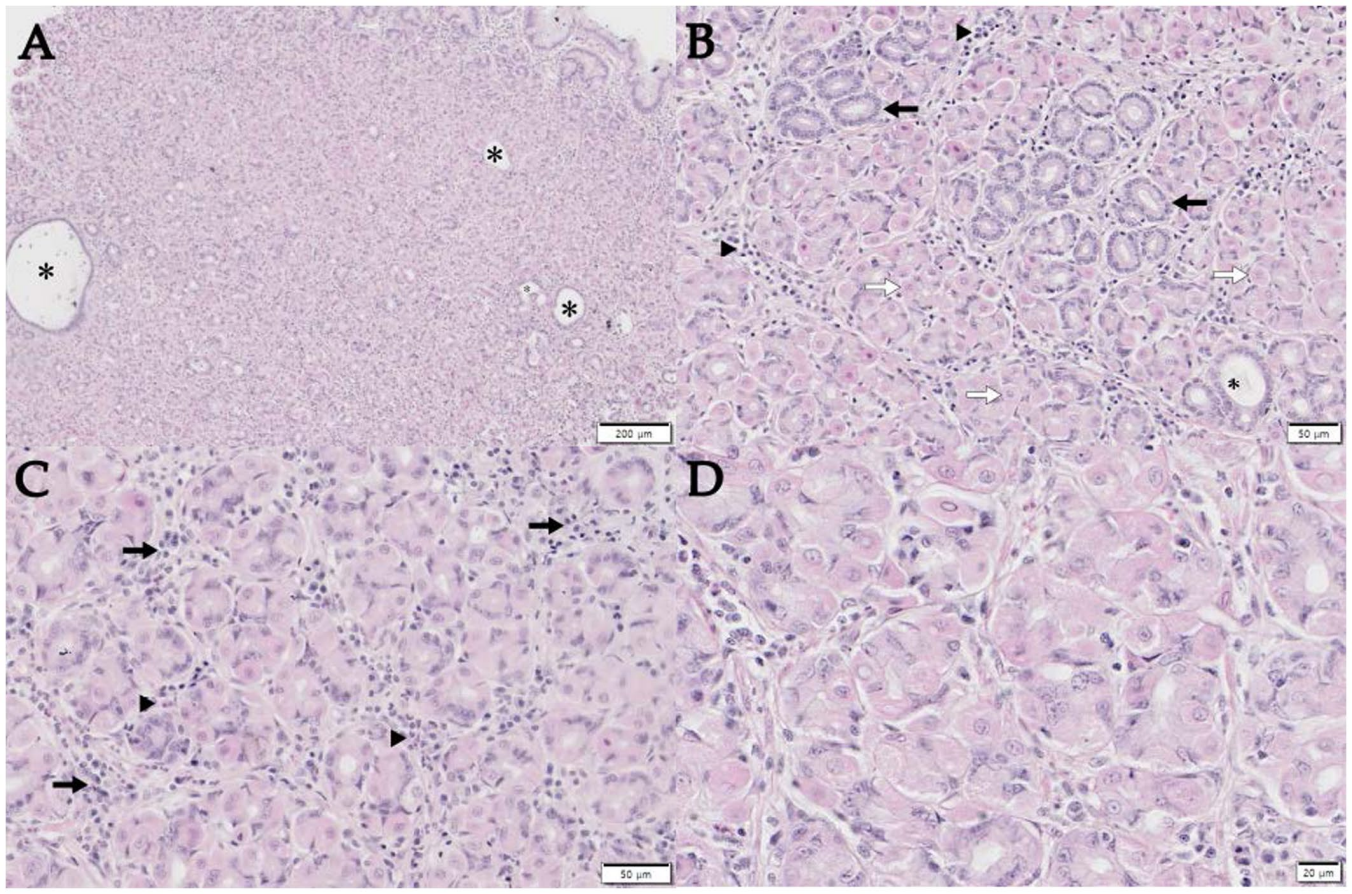
Histopathology of a punch biopsy of the thickened gastric mucosa. **(A)** Note the marked thickening of the mucosa due to the marked hyperplastic parietal cells, without foveolar hyperplasia, with occasional cystic dilatation of the glands (asterisk). Scale bar, 200 μm. **(B)** Diffuse parietal cell hyperplasia (open arrows) with reduced numbers of chief cells (arrows) is observed. Mild-to-moderate multifocal lymphoplasmacytic infiltration in the lamina propria (arrowheads) and gastric gland with cystic dilatation (*) are also noted. Scale bar, 50 μm. **(C)** The stroma contains multifocal lymphoplasmacytic infiltrates (arrows) with occasional eosinophils (arrowheads) in the lamina propria. Scale bar, 50 μm. **(D)** Hyperplastic parietal cells were pleomorphic and vacuolated in the cytoplasm. Hematoxylin and eosin staining, Scale bar, 20 μm.

Gastroscopy was performed to further investigate the abnormal findings detected on abdominal ultrasonography; this confirmed the presence of marked hypertrophy of the gastric folds and polypoid lesions (Figure 2). The large gastric folds extended across most of the gastric body and parts of the gastric fundus and could not be straightened even when the operator inflated the stomach. Polyps were observed throughout the gastric body. No erosions or ulcerations were observed. Biopsies of the polypoid lesions were obtained during endoscopy; histological examination revealed diffuse and marked parietal cell hyperplasia without foveolar hyperplasia, and occasional cystic dilatation of the gastric glands (Figure 3A). Prominent parietal cell hyperplasia with a decreased number of chief cells were observed (Figure 3B). The stroma contained multifocal inflammatory infiltrates including lymphocytes, plasma cells, and occasional eosinophils in the lamina propria (Figure 3C). Most of the hyperplastic parietal cells were pleomorphic with vacuolated cytoplasm (Figure 3D).

**Figure 4:**
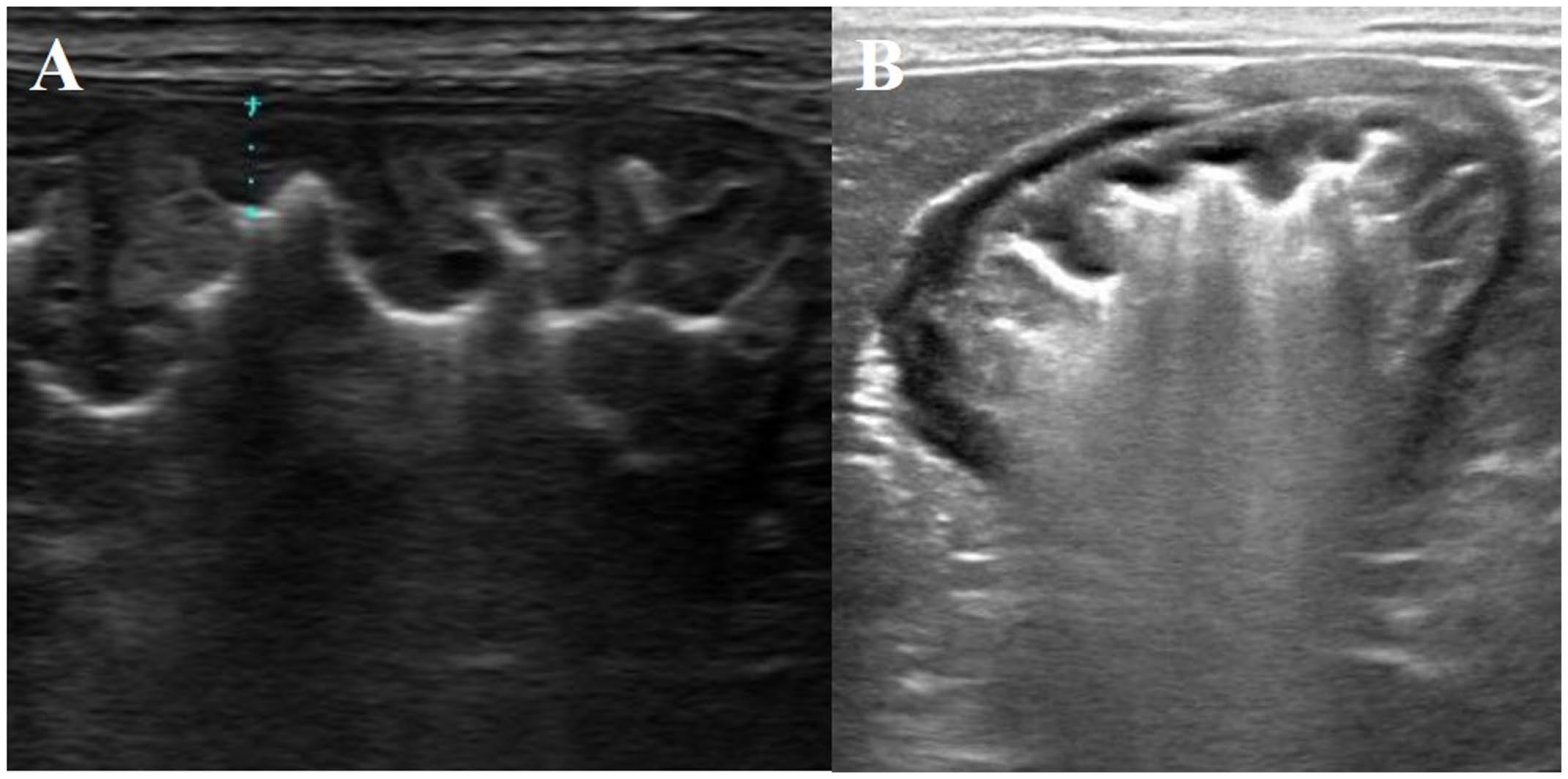
Ultrasonographic findings of the stomach, showing improvement and resolution of the gastric mucosal abnormalities 2 **(A)** and 48 months **(B)** after the discontinuation of omeprazole administration, respectively.

Therefore, omeprazole administration was discontinued, and prednisolone was administered (0.25 mg/kg PO q12 h), as previous reports had described hypertrophy of the gastric mucosa, manifesting as fundic gland and gastric hyperplastic polyps, in human patients who had been taking omeprazole for an extended period (10, 11). Furthermore, prednisolone could also reduce cerebrospinal fluid production. The serum gastrin concentration was measured using a [^125^I] radioimmunoassay and was shown to be high (78.4 ng/L; RI = 10–40 ng/L) (12). Within 1 month of discontinuing the omeprazole, the dog’s owner reported reduced frequency of vomiting. Two months after the discontinuation, vomiting had ceased and the dog showed improvement in the thickening of the gastric wall and folds, although this had not completely normalized (Figure 4A). Further endoscopic and histopathological assessments were not performed at the owner’s request. The dog remained alive without clinical signs such as vomiting 48 months after omeprazole administration was discontinued. At this time, the ultrasonographic findings of the stomach were not significant (Figure 4B), and the serum gastrin concentration was within RI (21.3 ng/L; RI = 10–40 ng/L).

## 3. Discussion

We describe a case of fundic gland polyps in a dog receiving long-term (> 4 years) omeprazole. This was evidenced by an improvement in clinical signs and resolution of the lesions after discontinuation of the drug. Histopathological findings of the lesions were marked parietal cell hyperplasia, without foveolar hyperplasia with occasional cystic dilatation of the gastric glands. This is consistent with the findings of fundic gland polyps in humans receiving omeprazole maintenance treatment (13). Long-term PPI administration can lead to hypertrophy of the gastric mucosa and the development of fundic glandular polyps in humans (10, 11).

Oral omeprazole administration increases gastric pH in dogs (14), which removes the inhibitory effect of gastric acid on G-cell secretion, further stimulating gastrin release (15). This leads to an even greater increase in gastric acid production by parietal cells, as in humans (11, 13, 16), and causes parietal cell hypertrophy and protrusion into the gland lumina, leading to their obstruction and cyst formation. Hypertrophic gastric polyps develop as the cysts enlarge (13).

Disorders with thickened gastric folds and polyps need to be differentially diagnosed based on their pathogenesis and histopathological characteristics. Menetrier’s-like disease is a condition that appears in the gastric bodies and fundus and is histologically characterized by foveolar hyperplasia and parietal atrophy in dogs (17, 18). *Helicobacter pylori* and cytomegalovirus infections are known to be related to Menetrier’s disease in humans and mice (19, 20), however, whether Menetrier’s-like disease is induced by PPI in dogs is unclear. Hypertrophic hypersecretory gastropathy is characterized by severe foveolar and parietal hyperplasia. However, our patient showed parietal hyperplasia without foveolar hyperplasia, and these histopathological differences helped differentiate and diagnose them despite their clinical and endoscopic similarities.

Some diseases cannot be distinguished endoscopically or histopathologically because of their similar characteristics. Zollinger–Ellison syndrome (ZES) has not only thickened gastric folds endoscopically but also parietal hyperplasia without foveolar hyperplasia histologically (21). ZES has been shown to be caused by a duodenal or pancreatic gastrinoma, which originates from G cells. Gastrin, which is abundantly released by gastrinomas, activates ECL cells, resulting in the synthesis and secretion of histamine. Gastrin and histamine stimulate gastric parietal cells to secrete hydrochloric acid and somatostatin from D cells for negative feedback, which is disrupted by gastrinoma, leading to ECL and parietal cell hyperplasia (22). Furthermore, most humans with ZES show gastric ulceration caused by severe hypergastrinemia (23). Gastric ulceration and tumor were absent in our patient, although the serum gastrin concentration was above the RI. Moreover, PPI can be an effective treatment for ZES in dogs (24). There was no evidence of gastrinoma on abdominal ultrasonography, and the patient survived for 4 years without any clinical signs of ZES.

The dog reported herein had been administered 0.7–1.0 mg/kg omeprazole orally every 24 h for over 4 years. There have been few descriptions of the long-term adverse effects of PPI administration in dogs, but diarrhea is the most commonly reported adverse effect in this species (25). Furthermore, the potential risk of hypergastrinemia, associated with the development of gastric tumors after long-term PPI administration, has been suggested in veterinary literature (3, 12). The dog reported herein had a high serum gastrin concentration although the concentration could not be directly compared with previously reported serum gastrin concentrations in healthy dogs (12), because the analyses were performed at different laboratories. Hypergastrinemia, secondary to the inhibition of acid secretion by omeprazole, might have been responsible for the fundic gland polyps in the present case, as in humans (13). The gastric lesions following PPI administration might be reversed after cessation of PPI administration (16), which occurred in our patient. The improvement in gastric lesions might have been secondary to the normalization of the serum gastrin concentration after the cessation of omeprazole administration; however, further studies are necessary to confirm that hypergastrinemia, secondary to omeprazole administration is associated with hypertrophic gastropathy in dogs.

Both the duration and dose of omeprazole treatment might be important in inducing fundic gland polyps. In human medicine, gastric adverse effects associated with long-term administration of PPI have been reported to generally occur after >1 year (10). However, no gastric changes were identified in a study in which dogs were administered omeprazole daily at 0.7 mg/kg for >1 year, and rugal hypertrophy similar to that of the present case was only identified in dogs that had been administered omeprazole at doses above 5.5 mg/kg/day (26). This suggests omeprazole might only induce fundic gland polyps in dogs when it is administered long-term (> 1 year) and at a high dose (> 5.5 mg/kg/day). The dog described herein had been administered 0.7–1.0 mg/kg omeprazole orally every 24 h for over 4 years, and the dose was gradually increased to 10 mg/kg orally every 24 h. To manage hydrocephalus, this dose was maintained for 8 months. Therefore, fundic gland polyps might be occurred in dogs receiving long-term high doses of omeprazole. Further studies are necessary to determine the duration and dosage of omeprazole administration at which fundic gland polyps might be induced.

In conclusion, we report a dog with fundic gland polyps that showed lesions similar to those of human patients receiving omeprazole for an extended period. Omeprazole is widely used for the long-term management of various diseases in dogs. Therefore, veterinarians should be aware of the adverse effects of long-term PPI administration, as described in the present case.

## Data availability statement

The raw data supporting the conclusions of this article will be made available by the authors, without undue reservation.

## Ethics statement

Ethical approval was not required for the studies involving animals in accordance with the local legislation and institutional requirements because the case report was a retrospective evaluation with no active interventional or research components. Written informed consent was obtained from the owners for the participation of their animals in this study.

## Author contributions

HL: Conceptualization, Formal analysis, Investigation, Methodology, Visualization, Writing – original draft. SaK: Formal analysis, Investigation, Methodology, Visualization, Writing – original draft. DL: Investigation, Methodology, Resources, Writing – review & editing. YC: Investigation, Methodology, Writing – review & editing. TY: Conceptualization, Writing – review & editing. M-PY: Conceptualization, Writing – review & editing. B-TK: Conceptualization, Writing – review & editing. SoK: Investigation, Methodology, Visualization, Writing – review & editing. HK: Conceptualization, Funding acquisition, Methodology, Supervision, Writing – review & editing.
